# Supplementary material for: The concentric β-barrel hypothesis for amyloids: Models of soluble and transmembrane amyloid-β42 oligomers and channels composed of identical subunits and GM1 gangliosides
Source: bioRxiv. 2026 Mar 23:2026.03.19.711324. Preprint. [Version 1] doi: 10.64898/2026.03.19.711324 (PMC13041797; doi:10.64898/2026.03.19.711324)
Supplement: Supplement 1 [file media-1.pdf]

SUPPLEMENT

The concentric β-barrel hypothesis for amyloids: Models of soluble and transmembrane amyloid-β42 oligomers and channels composed of identical subunits and GM1 gangliosides.

Stewart R. Durell[1], Yinon Shafrir[1], and H. Robert Guy[2*]

[1] Laboratory of Cell Biology, Bldg. 37 Rm 2108, National Cancer Institute, National Institutes of Health, Bethesda, MD 20892-4258, USA

[2]Amyloid Research Consultants (ARC), 6510 Tahawash Street, Cochiti Lake, NM 87083, USA

*Contact information:

H. Robert Guy

Amyloid Research Consultants (ARC),

6510 Tahawash Street,

Cochiti Lake, NM 87083, USA

Hrguy46@yahoo.com; (505-465-2445)

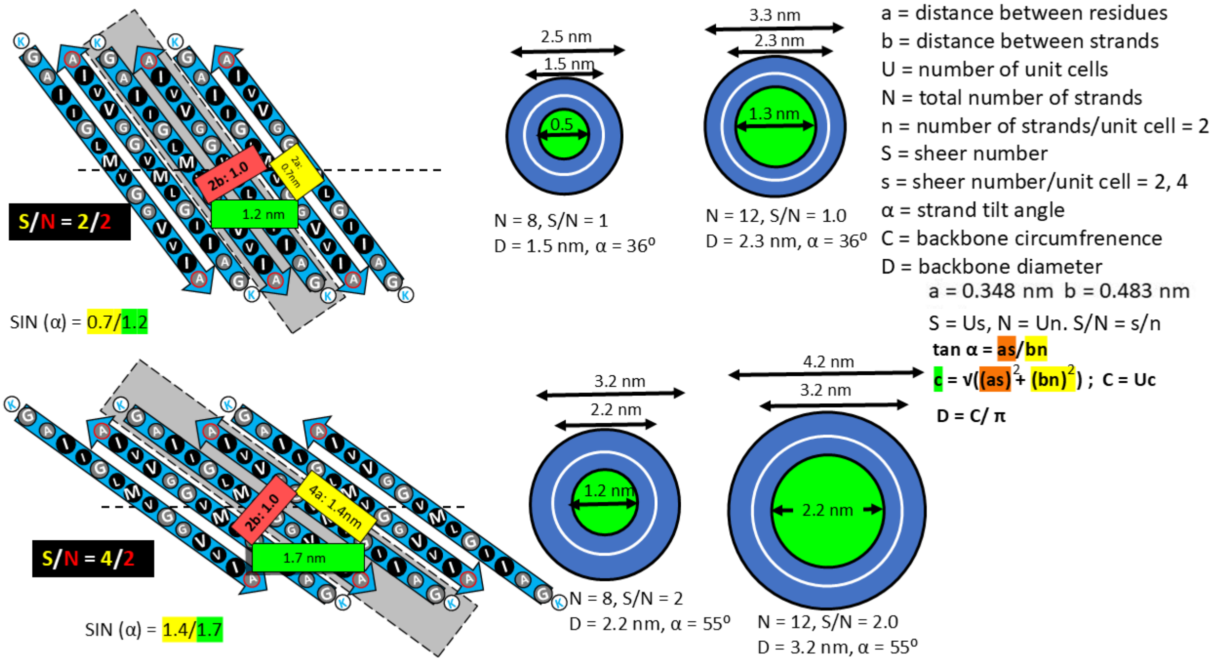

Figure S1. Beta barrel theory and definition of terms for 1Con models. The central unit cell in the flattened schematics has a gray background.

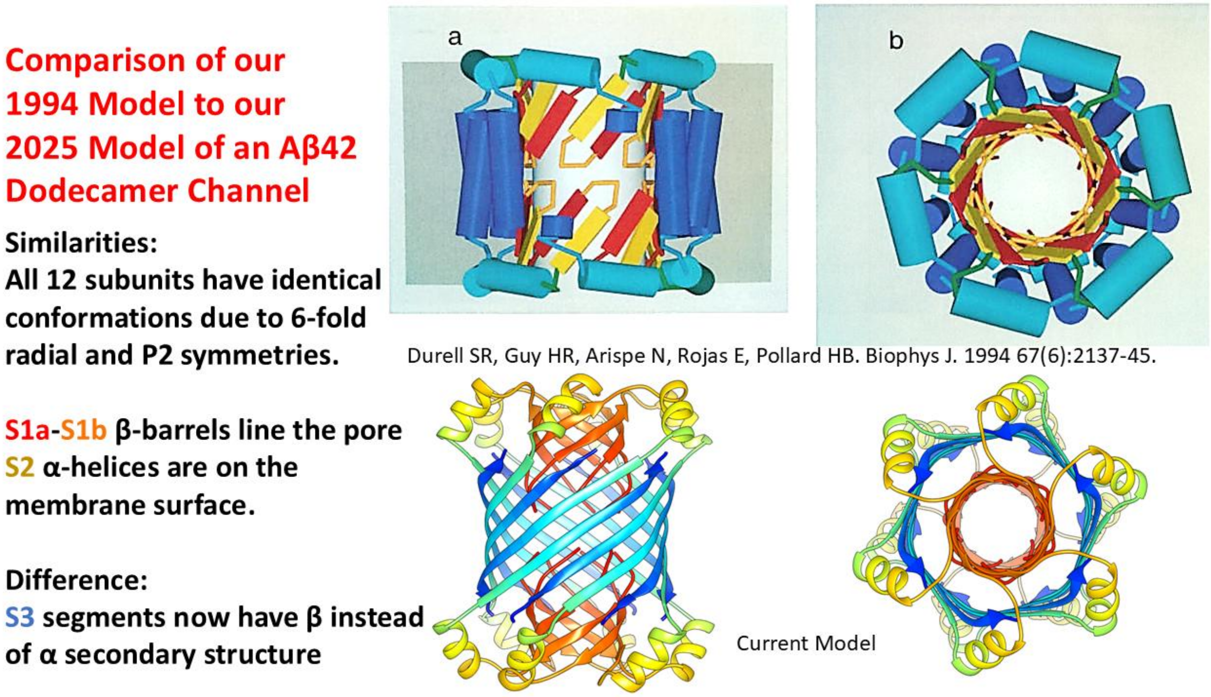

Figure S2. Comparison to first and current Aβ42 dodecamer channel models. (a & b) Top and side views of backbone. S2 helix is cyan in the ’94 model.
